# Rechute mammaire isolée d’un lymphome de Hodgkin: à propos d’un cas rare

**DOI:** 10.11604/pamj.2022.42.29.35083

**Published:** 2022-05-12

**Authors:** Najat Lasri, Fatimazahra Lahlimi, Mohammed Ilias Tazi

**Affiliations:** 1 Service d´Hématologie Clinique et de Greffe de Moelle, Centre Hospitalier Universitaire Mohammed VI, Faculté de Médecine et de Pharmacie, Université Cadi Ayyad, Marrakech, Maroc

**Keywords:** Néoplasie mammaire, lymphome de Hodgkin, traitement de rattrapage, cas clinique, Breast cancer, Hodgkin’s lymphoma, salvage therapy, case report

## Abstract

La localisation mammaire isolée d´un lymphome de Hodgkin est rare. Elle peut prêter à confusion avec les autres néoplasies et affections inflammatoires mammaires. Nous rapportons le cas d´une patiente âgée de 18 ans traitée pour lymphome de Hodgkin à cellularité mixte, qui s´est présenté 6 mois après la rémission complète pour un nodule mammaire inflammatoire gauche. Le bilan anatomopathologique initial a été en faveur d´un abcès mammaire. Les investigations face à l´évolution défavorable malgré une antibiothérapie adaptée ont été en faveur d´une rechute du LH avec une localisation mammaire isolée. La patiente est actuellement sous 4ème ligne thérapeutique (polychimiothérapie). Les lymphomes Hodgkiniens mammaires sont connus par leur pronostic défavorable. Les progrès thérapeutiques (thérapie ciblée) peuvent améliorer le devenir des patientes.

## Abstract (en)

Relapsed Hodgkin lymphoma with only breast involvement is rare. There is no specific clinical presentation. It can mimic another breast cancer or inflammatory disease. The histological profile is often misleading in the pauci-cellular forms where the inflammatory component predominates. We report a case of an 18-year-old female patient treated for mixed-cell Hodgkin lymphoma who was admitted 6 months after complete remission for a left breast inflammatory nodule. The first assessment showed breast abscess. As there was not a good outcome despite adapted antibiotic, more investigations were added and showed a relapsed Hodgkin lymphoma with only breast involvement. The patient is currently receiving a 4^th^ therapeutic line regimen of chemotherapy. Breast Hodgkin lymphoma´s prognosis is poor in most cases reported in the literature. Therapeutic advances (targeted therapy) can improve patient outcomes.

## Introduction

La localisation mammaire isolée du lymphome de Hodgkin est une entité rare. Les tableaux clinique et radiologique ne sont pas spécifiques [1], le risque de confusion avec les autres cancers et les mastopathies inflammatoires bénignes du sein constitue un piège diagnostique [1,2]. Nous rapportons le cas d´une rechute mammaire isolée d´un lymphome de Hodgkin chez une patiente âgée de 18 ans.

## Patient et observation

**Informations du patient**: une patiente âgée de 18 ans, ayant comme antécédant un lymphome de Hodgkin à cellularité mixte stade IV par le poumon traité par chimiothérapie à base du protocole OPPA-COPP complété par radiothérapie. Elle s´est présenté 6 mois après la rémission complète pour un nodule inflammatoire mammaire gauche, sans autres symptômes associés.

**Résultats cliniques**: l´examen clinique a trouvé un performens status à 0, un nodule mammaire du quadrant supéro-externe de 2cm /2cm, inflammatoire, chaud et douloureux, sans adénopathies périphériques ni autres anomalies associés.

**Figure 1:**
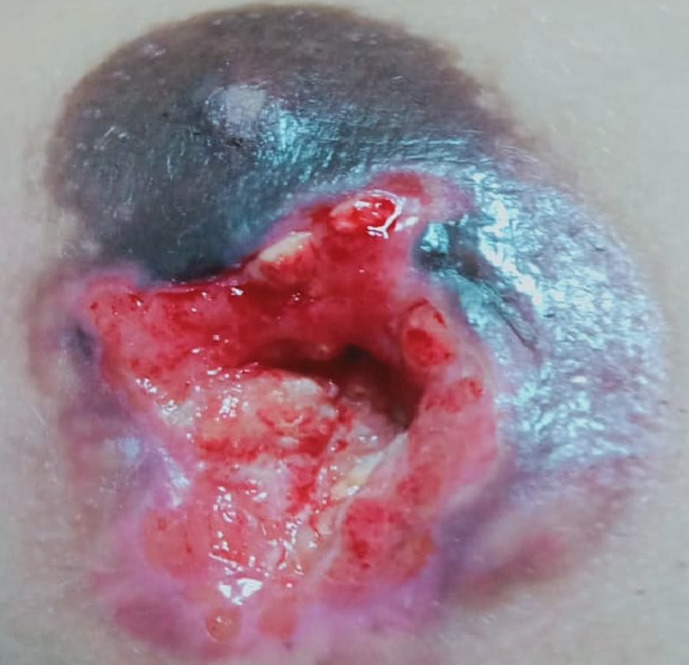
ulcération mammaire gauche ayant révélé la rechute du lymphome de Hodgkin avec une localisation mammaire isolée

**Démarche diagnostique**: l´échographie mammaire a objectivé une plage hypoéchogène hétérogène du quadrant supéro-externe gauche avec discrets signes d´inflammation. Deux biopsies successives du nodule, à un mois d´intervalle, ont été en faveur d´un remaniement inflammatoire en poussée avec singes d´abcédation, sans signes de malignité. Devant la suspicion d´abcès mammaire, notre patiente a été mise sous antibiothérapie à double reprises à base d´amoxicilline-acide clavulanique sans amélioration, avec apparition d´une ulcération mammaire d´extension rapidement progressive (Figure 1). Cette lésion mammaire n´est pas spécifique du lymphome de Hodgkin, elle peut se voir dans les autres mastopathies notamment infectieuses et cancéreuses. Dans ce contexte, une 3^e^ biopsie mammaire a été faite qui a révélé la rechute mammaire du lymphome de Hodgkin, avec à l´examen histologique et immunohistochimique: une mastite granulomonateuse, présence de nombreuses cellules tumorales Sternbergoides et typiques Hodgkinienne, CD 15 +, CD 20 +, PAX-5+. Les LDH étaient élevés à 2 fois la normale. La numération sanguine n´a pas montré de particularités à part une anémie microcytaire à 11,5g/l. la VS ainsi que le taux d´albumine ont été normaux. La TDM-TAP dans le cadre du bilan d´extension a objectivé une localisation mammaire isolée sous forme d´un processus lésionnel mal limité rehaussé de façon hétérogène après injection de produit de contraste. La BOM n´a pas montré d´envahissement médullaire. Le diagnostic de rechute mammaire d´un lymphome de Hodgkin à cellularité mixte groupe haut risque a été posé.

**Intervention thérapeutique**: la patiente a été incluse dans un protocole de rattrapage à base de ICE (ifosfamide, carboplatine, étoposide), avec autogreffe des SCH envisageable. L´évolution a été marquée par une progression de maladie après 3 lignes thérapeutique (ICE puis DHAP: aracytine, carboplatine,déxaméthasone puis GEMOX: gemcitabine-oxaliplatine ) avec présence au PET scanner d´évaluation après chaque ligne d´un processus mammaire hypermétabolique deauville V. Notre patiente est actuellement sous 4^e^ ligne thérapeutique par le protocole BeGVD (bendamustine – gemcitabine - vinorelbine-doxirubicine). Une autogreffe des cellules souches hématopoïétiques sera réalisée dans le cas où une rémission complète sera obtenue.

**Suivi et résultats**: notre patiente est actuellement sous 4^e^ ligne thérapeutique par le protocole BeGVD (bendamustine - gemcitabine - vinorelbine-doxirubicine). Une autogreffe des cellules souches hématopoïétiques sera réalisée dans le cas où une rémission complète sera obtenue.

## Discussion

Les lymphomes mammaires ne représentent que 0,5% des cancers du sein [1]. La localisation mammaire isolée d´un lymphome de Hodgkin, initiale ou en rechute est extrêmement rare. Peu de données notamment épidémiologiques existent dans la littérature vu l´extrême rareté des séries. La localisation mammaire isolée du lymphome de Hodgkin prête souvent à confusion avec les autres néoplasies et affections inflammatoires mammaires. Cliniquement, le motif de consultation le plus fréquent et l´apparition d´un nodule mammaire non inflammatoire [1-3] contrairement à notre cas où l´état inflammatoire clinique a été très manifeste.

Les données de mammographie et d´échographie mammaires ne sont pas pathognomoniques (lésion hyperdense dans 81% des cas dans la mammographie). La biopsie chirurgicale est nécessaire au diagnostic, la cytoponction est non concluante dans la majorité des cas, contrairement aux autres cancers mammaires [4]. La description anatomopathologique est souvent trompeuse dans les formes paucicellulaires où la composante inflammatoire prédomine comme est le cas chez notre patiente. Elles causent souvent une errance diagnostique car elles peuvent mimer un carcinome canalaire, ou une autre mastopathie bénigne [3]. L´immunohistochimie montre la positivité des marqueurs CD 30, CD 15 et PAX5, avec négativité de cytokératine qui permet l´exclusion des autres cancers mammaires [5]. L´IRM mammaire reste pratiquée dans certains centres, mais le PET scanner demeure l´examen performant dans le cadre du bilan lésionnel et d´évaluation. Le degré d´hypermétabolisme au PET scanner dans le lymphome de Hodgkin mammaire n´est pas différent des autres mastopathies malignes [3].

Le traitement du lymphome de Hodgkin en rechute avec localisation mammaire isolée ne diffère pas des autres formes cliniques du lymphome de Hodgkin en rechute, qui se base sur l´association de chimiothérapie de 2^e^ ligne (ICE: ifosfamide, carboplatine, étoposide ou DHAP: aracytine haute dose, carboplatine, dexamethasone) avec l´anticorps monoclonal anti-CD 30 (brentuximab védotin), suivie d´intensification thérapeutique par le protocole BEAM (carmustine, étoposide, aracytine, melphalan) et d´autogreffe des cellules souches hématopoïétique [6-8]. L´efficacité d´un traitement d´entretien par le brentixumab védotin dans les groupes de risque élevé a été prouvé par certains auteurs. Dans les formes réfractaires à plusieurs lignes thérapeutiques, le projet d´allogreffe peut être discuté [9]. Le traitement chirurgical n´a pas de place [1]. Le pronostic de rechute mammaire de lymphome de Hodgkin est médiocre dans la plupart des cas rapportés dans la littérature sous forme de case report, avec un caractère majoritairement réfractaire comme est le cas chez notre patiente [1].

**Point de vue de la patiente**: au décours du traitement, la patiente n´a pas ressenti une amélioration.

**Consentement éclairé**: nous avons obtenu le consentement éclairé de la patiente pour utiliser les images dans ce rapport de cas.

## Conclusion

La localisation mammaire isolée du lymphome de Hodgkin en rechute est rare. Le diagnostic risque d´être masqué par des présentations cliniques et histologiques trompeuses. Peu de données de littérature sont disponibles concernant la prise en charge et le pronostic global liés à cette entité vu la rareté des séries.
